# Supplementary material for: Tuberculosis severity associates with variants and eQTLs related to vascular biology and infection-induced inflammation
Source: PLoS Genet. 2023 Mar 27;19(3):e1010387. doi: 10.1371/journal.pgen.1010387 (PMC10079228; doi:10.1371/journal.pgen.1010387)
Supplement: S1 Methods — (PDF) [file pgen.1010387.s001.pdf]

Supplemental Methods

TBscore

The TBscore is based on five self-reported symptoms: cough, hemoptysis, dyspnea, chest pain, and night sweats, as well as six traits measured at examination: anemia, pulse > 90 beats/min, positive findings at lung auscultation, temperature > 37 ° C, body mass index (BMI) < 18 kg/m^2, and mid upper arm circumference (MUAC) < 220 mm. Each of the 11 clinical variables contributes 1 point, while BMI and MUAC can contribute an extra point if <16 kg/m 2 and < 200 mm, respectively; thus, the maximum TBscore is 13 (S1 Table). In this study, we did not have data on MUAC, so instead used lean and fat mass body composition data obtained using bioelectrical impedance analysis (BIA), as described previously (1). Overall, the TBscore provides information on clinical course, prognostic value, biologically meaningful biomarkers, and patient experience. Thus, the TBscore is a valid clinical measure that assesses patients' severity as well as indicating prognosis.

GWAS Quality Control (QC)

The genotype data for Cohort 1 initially included 2,036,060 SNPs. After QC and prior to imputation, there were 717,705 SNPs remaining. After imputation and QC, we had 8,146,092 SNPs (S1 Fig). The genotype data for Cohort 2 included 2,989,642 SNPs initially. After QC, 1,931,961 SNPs remained prior to imputation. After imputation and another round of QC thresholds, there were 9,626,100 SNPs (S1 Fig). These were the final numbers of SNPs in each cohort prior to meta-analysis.

Principal Component Computation and Analysis

We computed principal components (PC's) but did not include these PC's in the regression analyses as the sample populations did not appear to show substantial substructure. This is consistent with previous studies that have shown there is little to no discernable genetic sub-structure within our study population based on microsatellite analyses and previous regression and PC analyses (2). Our decision was based on 3 pieces of evidence in our data that we used to decide against their inclusion *a priori*: 1.) There did not appear to be clustering in the plot of PC1 vs. PC2 (S2 and S3 Figs); 2.) Based on the eigenvalues, the PC's did not explain a substantial amount of variation in the genotypes of the subjects included in the cohort (2.8% for the top PC in Cohort 1 and 4.3% for the top PC in Cohort 2); and 3.) the top 3 PC's did not associate with the outcome (i.e. TBscore). The P-value for the association between PC1 and TBscore was 0.2 in Cohort 1 and 0.6 in Cohort 2, using a linear regression model (and these were the lowest P-values for any of the top 10 PC's in either cohort). If the PC's neither correlate with the outcome nor explain variation in the genotype data, then it is unlikely that unmeasured population substructure will bias the association between genotype and TBscore. However, we also performed a sensitivity analysis to determine how the inclusion of PC's in the regression equations for each cohort would affect the P-values and Beta coefficients for the top SNP.

Enrichment and Annotation of Severity Associated SNPs

In order to provide greater depth of meaning and interpretability to our GWAS summary statistic results, we utilized FUMA GWAS to provide annotation of individual SNPs, such as regulatory information, tissue specificity, and gene mapping (3). We utilized $p<1e-05$ as the threshold for enrichment and annotation and a distance of +/- 10 kb for gene mapping to assign a gene to SNPs that were not located within a gene. We also utilized FUMA GWAS'

GENE2FUNC feature for the genes represented in the GWAS summary statistics. This feature maps GWAS summary statistics to genes, and then provides gene set or pathway enrichments based on gene sets from MsigDB, KEGG, WikiPathways, and the GWAS Catalog. In order to determine significance, FUMA uses hypergeometric mean pathway analysis and adjusts the p-values for significance based on the Benjamini-Hochberg method (i.e. FDR) using the number of data sources of tested gene sets as the number of independent tests performed in the multiple testing correction. FUMA reports gene sets with adjusted P-value ≤ 0.05 and the number of genes that overlap with the gene set > 1 (3). In addition to FUMA, we utilized GeneCards, Ensembl, DICE, and STRING DB to help annotate and enrich our results with respect to function, expression, and downstream protein interactions (4-7).

Another feature that FUMA provides is a MAGMA gene-based analysis using the full range of GWAS summary statistics. MAGMA is a computational biology tool that utilizes the significance and direction of effect of multiple SNPs aggregated together in the same genes (8). MAGMA is “based on a multiple linear principal components regression model, using an F-test to compute the gene p-value” (8). In addition to looking for gene-level associations, FUMA also used MAGMA’s gene set enrichment and tissue specificity analyses, the latter of which uses tissue-specific data on gene expression from GTEx v8. When uploading summary statistics to FUMA, it automatically runs the MAGMA analyses on the data, provided that summary statistics for all SNPs tested are uploaded. Thus, it helps inform the tissue or pathway specific functions of the gene-level associations, rather than mapping individual SNPs to genes and using those. We also decided to look up SNPs in the region of genes that have previously been associated with TB susceptibility. This allows for a comparison of the determinants of each phenotype and can demonstrate whether or not there are different genes associated with each

phenotype. To accomplish this, we pulled the genes from a previous review by Stein et al. and examined SNPs within a +/- 5kb window of the gene (9).

RNA-Seq Methods

For the purpose of measuring gene expression in the 72 RSTR and LTBI subjects for our eQTL analyses, RNA sequencing was performed on the Illumina HiSeq 2500 in high output mode, which achieved a read depth of 30 million paired-end, 50 base pair reads. Adapters were removed prior to sequence alignment using STAR 2.6.0 (10). Counts for each gene were assigned using RSEM 1.3.0 (11). Using multidimensional scaling (MDS), we found no evidence of batch effects nor any global influence of age or the other available metadata from each cohort (12).

After transcript reads were mapped to genes and pooled gene counts were generated, we filtered the mapped genes to exclude genes not contained on chromosome 1-22 and non-protein coding genes. We limited the data to non-protein coding genes so we could observe changes in expression of genes that had functional downstream consequences. DNA variants in non-coding regions were still examined for regulatory effects but we did not examine changes in expression for non-coding genes. Then, the pooled counts were normalized using the trimmed mean of M-values method, which reduces the possibility of bias due to read depth and variations in library size (13). This step is necessary to allow for direct and unbiased comparison of expression across different genes. After normalization, genes were further filtered out if they had low or no expression across all samples. After QC and filtering, there were 13,547 genes remaining in this analysis.

eQTL analysis

Matrix eQTL tests cis and trans eQTL's separately and the subsequent FDR correction used to determine significance is performed separately for each category. In this analysis, we

defined a cis eQTL as being within 500Kb of the gene location downloaded from Biomart in Ensembl Version 104 using the GRCh37 (Hg19) human genome build (14). We generated the list of differentially expressed genes using the tag-wise dispersion method (15). A log2 fold change greater than 1 or less than -1 (i.e. genes with expression levels that either double or halved after stimulation with MTB) and an FDR of 0.01 was used as the definition of differential expression. This list of differentially expressed genes was then used to test for eQTL's. All QC, filtering, and differential analyses were performed using Limma, Voom, and EdgeR packages in the Bioconductor suite for R v3.6.3 (16-19).

The final study population for our follow-up eQTL analysis included 72 subjects without active TB that had data for RNA expression and genotype (S5 Table). The differential expression analysis yielded 1,986 genes that met the definition for differential expression (S6 Table). 161 of our severity-associated SNPs were also available for analysis in the genotype chip. In total, there 78 cis SNP-gene pairings and 332,065 trans SNP- gene pairings, where a cis pairing represented any combination of a SNP and a gene located within 500 kb and a trans pairing represented any combination of a SNP and a gene greater than 500 kb from the target gene.

One of these was rs7707024, a G to C change at position 159320915 on chromosome 5 that associated with a 1-point reduction in TBscore (P=0.02, β= -0.93). rs7707024 is a non-coding transcript variant in a long non-coding RNA (lncRNA) gene, *LINC01847*. rs7707024 is in a known regulatory region according to Ensembl and is suggested to be an enhancer, but this has not been experimentally verified. We also identified a second SNP, rs13358509, which is an intron variant for *LOC285626*, an uncharacterized novel transcript. rs13358509 associated with a 0.71 reduction in TBscore (P=0.039).

Cited References

1. Fluegge K, Malone LL, Nsereko M, Okware B, Wejse C, Kisingo H, et al. Impact of geographic distance on appraisal delay for active TB treatment seeking in Uganda: a network analysis of the Kawempe Community Health Cohort Study. BMC public health. 2018;18(1):798.
2. McHenry ML, Bartlett J, Igo RP, Jr., Wampande EM, Benchek P, Mayanja-Kizza H, et al. Interaction between host genes and Mycobacterium tuberculosis lineage can affect tuberculosis severity: Evidence for coevolution? PLoS genetics. 2020;16(4):e1008728.
3. Watanabe K, Taskesen E, van Bochoven A, Posthuma D. Functional mapping and annotation of genetic associations with FUMA. Nature Communications. 2017;8(1):1826.
4. von Mering C, Jensen LJ, Snel B, Hooper SD, Krupp M, Foglierini M, et al. STRING: known and predicted protein-protein associations, integrated and transferred across organisms. Nucleic acids research. 2005;33(Database issue):D433-7.
5. Stelzer G, Rosen N, Plaschkes I, Zimmerman S, Twik M, Fishilevich S, et al. The GeneCards Suite: From Gene Data Mining to Disease Genome Sequence Analyses. Current Protocols in Bioinformatics. 2016;54(1):1.30.1-1..3.
6. Ha B, Greenbaum JA, Shmiedel BJ, Singh D, Madrigal A, Valdovino-Gonzalez AG, et al. Database of Immune Cell EQTLs, Expression, Epigenomics. The Journal of Immunology. 2019;202(1 Supplement):131.18-.18.
7. Flicek P, Amode MR, Barrell D, Beal K, Brent S, Chen Y, et al. Ensembl 2011. Nucleic acids research. 2011;39(Database issue):D800-D6.
8. de Leeuw CA, Mooij JM, Heskes T, Posthuma D. MAGMA: generalized gene-set analysis of GWAS data. PLoS computational biology. 2015;11(4):e1004219.
9. Stein CM, Sausville L, Wejse C, Sobota RS, Zetola NM, Hill PC, et al. Genomics of human pulmonary tuberculosis: from genes to pathways. Curr Genet Med Rep. 2017;5(4):149-66.
10. Dobin A, Davis CA, Schlesinger F, Drenkow J, Zaleski C, Jha S, et al. STAR: ultrafast universal RNA-seq aligner. Bioinformatics. 2013;29(1):15-21.
11. Li B, Dewey CN. RSEM: accurate transcript quantification from RNA-Seq data with or without a reference genome. BMC Bioinformatics. 2011;12:323.
12. Simmons JD, Van PT, Stein CM, Chihota V, Ntshiqa T, Maenetje P, et al. Monocyte metabolic transcriptional programs associate with resistance to tuberculin skin test/interferon-γ release assay conversion. The Journal of clinical investigation. 2021;131(14).
13. Robinson MD, Oshlack A. A scaling normalization method for differential expression analysis of RNA-seq data. Genome Biol. 2010;11(3):R25.
14. Cunningham F, Achuthan P, Akanni W, Allen J, Amode MR, Armean IM, et al. Ensembl 2019. Nucleic acids research. 2019;47(D1):D745-D51.
15. Soneson C, Delorenzi M. A comparison of methods for differential expression analysis of RNA-seq data. BMC Bioinformatics. 2013;14(1):91.
16. Law CW, Chen Y, Shi W, Smyth GK. voom: Precision weights unlock linear model analysis tools for RNA-seq read counts. Genome biology. 2014;15(2):1-17.
17. Ritchie ME, Phipson B, Wu D, Hu Y, Law CW, Shi W, et al. limma powers differential expression analyses for RNA-sequencing and microarray studies. Nucleic acids research. 2015;43(7):e47-e.
18. Robinson MD, McCarthy DJ, Smyth GK. edgeR: a Bioconductor package for differential expression analysis of digital gene expression data. Bioinformatics. 2010;26(1):139-40.
19. Gentleman RC, Carey VJ, Bates DM, Bolstad B, Dettling M, Dudoit S, et al. Bioconductor: open software development for computational biology and bioinformatics. Genome biology. 2004;5(10):1-16.
