# Supplementary material for: Tuberculosis severity associates with variants and eQTLs related to vascular biology and infection-induced inflammation
Source: PLoS Genet. 2023 Mar 27;19(3):e1010387. doi: 10.1371/journal.pgen.1010387 (PMC10079228; doi:10.1371/journal.pgen.1010387)
Supplement: S1 Table — (DOCX) [file pgen.1010387.s002.docx]

**Table S1. Components of the TBscore and Points Contributed to Final Score**

| **Symptom** | **Points** |
| --- | --- |
| Cough | 1 |
| Hemoptysis | 1 |
| Dyspnea | 1 |
| Chest Pain | 1 |
| Night Sweats | 1 |
| Anemia | 1 |
| Pulse > 90 BPM | 1 |
| Lung auscultation (+) | 1 |
| Temperature > 37 ° C | 1 |
| BMI < 18 kg/m^2 | 1 |
| MUAC < 220 mm | 1 |
| BMI <16 kg/m^2 | 1 |
| MUAC < 200 mm | 1 |
