# Supplementary material for: Tuberculosis severity associates with variants and eQTLs related to vascular biology and infection-induced inflammation
Source: PLoS Genet. 2023 Mar 27;19(3):e1010387. doi: 10.1371/journal.pgen.1010387 (PMC10079228; doi:10.1371/journal.pgen.1010387)
Supplement: S2 Table — (DOCX) [file pgen.1010387.s003.docx]

**Table S2. Studies of eQTL’s and Gene Expression**

| **Findings** | **Population** | **Phenotype** | **Citation** |  |
| --- | --- | --- | --- | --- |
| **Differential Expression Studies** | | | | |
| *FCGRIB* | South Africa | TB vs. LTBI & Controls | (1) |  |
| *CCL1* | Vietnam | Pulmonary vs. Meningeal & Latent | (2) |  |
| *IL1B*, *IL2*, and *TNF* | European | MTB-stimulated *in vitro* | (3) |  |
| Type 1 IFN's | USA | Stimulation with MTB vs. other pathogens *in vitro* | (4) |  |
| iNOS, GTPases, protease inhibitors, chemokines, and chemokine receptors | Mice | Stimulation with MTB and IFN-γ | (5) |  |
| *IL12B* | USA | Stimulation with MTB vs. other pathogens *in vitro* | (6) |  |
| **eQTL Studies** | | | | |
| rs9828868~*ZXDC* | France | Levels of IFN-γ after stimulation | (7) |  |
| List of DE Genes | USA | MTB-stimulated *in vitro* | (8) |  |
| rs62292160~*IL4* | China (Han) | Active TB vs. LTBI | (9) |  |
| List of DE Genes |  | MTB-stimulated *in vitro* | (10) |  |

1. Maertzdorf J, Repsilber D, Parida SK, Stanley K, Roberts T, Black G, et al. Human gene expression profiles of susceptibility and resistance in tuberculosis. *Genes & Immunity.* 2011;12(1):15-22.

2. Thuong NTT, Dunstan SJ, Chau TTH, Thorsson V, Simmons CP, Quyen NTH, et al. Identification of Tuberculosis Susceptibility Genes with Human Macrophage Gene Expression Profiles. *PLOS Pathogens.* 2008;4(12):e1000229.

3. Ragno S, Romano M, Howell S, Pappin DJ, Jenner PJ, and Colston MJ. Changes in gene expression in macrophages infected with Mycobacterium tuberculosis: a combined transcriptomic and proteomic approach. *Immunology.* 2001;104(1):99-108.

4. Chaussabel D, Semnani RT, McDowell MA, Sacks D, Sher A, and Nutman TB. Unique gene expression profiles of human macrophages and dendritic cells to phylogenetically distinct parasites. *Blood.* 2003;102(2):672-81.

5. Ehrt S, Schnappinger D, Bekiranov S, Drenkow J, Shi S, Gingeras TR, et al. Reprogramming of the macrophage transcriptome in response to interferon-γ and Mycobacterium tuberculosis: signaling roles of nitric oxide synthase-2 and phagocyte oxidase. *The Journal of experimental medicine.* 2001;194(8):1123-40.

6. Nau GJ, Richmond JF, Schlesinger A, Jennings EG, Lander ES, and Young RA. Human macrophage activation programs induced by bacterial pathogens. *Proceedings of the National Academy of Sciences.* 2002;99(3):1503-8.

7. Jabot-Hanin F, Cobat A, Feinberg J, Orlova M, Niay J, Deswarte C, et al. An eQTL variant of ZXDC is associated with IFN-γ production following Mycobacterium tuberculosis antigen-specific stimulation. *Sci Rep.* 2017;7(1):12800.

8. Barreiro LB, Tailleux L, Pai AA, Gicquel B, Marioni JC, and Gilad Y. Deciphering the genetic architecture of variation in the immune response to <em>Mycobacterium tuberculosis</em> infection. *Proceedings of the National Academy of Sciences.* 2012;109(4):1204-9.

9. Ai J-W, Zhang H, Zhou Z, Weng S, Huang H, Wang S, et al. Gene expression pattern analysis using dual-color RT-MLPA and integrative genome-wide association studies of eQTL for tuberculosis suscepitibility. *Respiratory Research.* 2021;22(1):23.

10. Blischak JD, Tailleux L, Myrthil M, Charlois C, Bergot E, Dinh A, et al. Predicting susceptibility to tuberculosis based on gene expression profiling in dendritic cells. *Scientific Reports.* 2017;7(1):5702.
