# Supplementary material for: Tuberculosis severity associates with variants and eQTLs related to vascular biology and infection-induced inflammation
Source: PLoS Genet. 2023 Mar 27;19(3):e1010387. doi: 10.1371/journal.pgen.1010387 (PMC10079228; doi:10.1371/journal.pgen.1010387)
Supplement: S6 Table — (DOCX) [file pgen.1010387.s007.docx]

**Table S6. MAF, P-value, and β value for rs1848553 Across Cohorts 1 and 2**

| **Cohort** | **MAF** | **P** | **β** |
| --- | --- | --- | --- |
| Cohort 1 | 25.10% | 2.81E-05 | -1.05 |
| Cohort 2 | 23.70% | 5.68E-04 | -0.89 |
| Meta | 24.70% | 2.97E-08 | -0.97 |

Minor allele frequencies in the 1000G project were ascertained from Ensembl Genome Browser v104
