# Supplementary material for: Tuberculosis severity associates with variants and eQTLs related to vascular biology and infection-induced inflammation
Source: PLoS Genet. 2023 Mar 27;19(3):e1010387. doi: 10.1371/journal.pgen.1010387 (PMC10079228; doi:10.1371/journal.pgen.1010387)
Supplement: S7 Table — (DOCX) [file pgen.1010387.s008.docx]

**Table S7. Allele Frequencies for rs1848553 in 1000G Project in African Populations and Non-African Super-Populations**

| **Population** | **Allele Frequency** |
| --- | --- |
| **African** | **C: 78.7% T: 21.3%** |
| ACB | C: 81.2% T: 18.8% |
| ASW | C: 84.4% T: 15.6% |
| ESN | C: 80.8% T: 19.2% |
| GWD | C: 83.2% T: 16.8% |
| LWK | C: 77.8% T: 22.2% |
| MSL | C: 74.7% T: 25.3% |
| YRI | C: 70.8% T: 29.2% |
| **American** | **C: 97.4% T: 2.6%** |
| **East Asian** | **C: 100%** |
| **European** | **C: 99.9% T: 0.01%** |
| **South Asian** | **C: 100%** |

Minor allele frequencies in the 1000G project were ascertained from Ensembl Genome Browser v104
