# Supplementary material for: Tuberculosis severity associates with variants and eQTLs related to vascular biology and infection-induced inflammation
Source: PLoS Genet. 2023 Mar 27;19(3):e1010387. doi: 10.1371/journal.pgen.1010387 (PMC10079228; doi:10.1371/journal.pgen.1010387)
Supplement: S10 Table — The genes analyzed are from a prior review paper by Stein et al. A +/- 5kb window around each gene was interrogated for potential associations with TBscore and the SNP with the lowest P-value in each window is shown in the table. (DOCX) [file pgen.1010387.s011.docx]

**Table S10. Association with TBscore for SNPs near Genes Previously Associated with TB Susceptibility.** The genes analyzed are from a prior review paper by Stein et al. A +/- 5kb window around each gene was interrogated for potential associations with TBscore and the SNP with the lowest P-value in each window is shown in the table.

| **CHR** | **BP** | **SNP** | **Effect Allele** | **P** | **BETA** | **Gene** |
| --- | --- | --- | --- | --- | --- | --- |
| 12 | 48317832 | rs7311030 | C | 0.009 | 0.46 | *VDR* |
| 12 | 68545664 | rs80057724 | A | 0.021 | -0.39 | *IFNG* |
| 9 | 120468649 | rs12344353 | C | 0.023 | 0.55 | *TLR4* |
| 2 | 219249013 | rs10762883 | T | 0.053 | 0.57 | *SLC11A1* |
| 6 | 137539737 | rs17175260 | C | 0.088 | 0.33 | *IFNGR1* |
| 4 | 154629838 | rs10034134 | G | 0.116 | 0.26 | *TLR2* |
| 10 | 54520749 | rs10762883 | G | 0.116 | 0.29 | *MBL2* |
| 3 | 52268866 | rs353547 | T | 0.121 | -0.26 | *TLR9* |
| 6 | 31550452 | rs3093551 | G | 0.134 | -0.72 | *TNF* |
| 17 | 32580820 | rs3091332 | T | 0.135 | -0.34 | *MCP1* |
| 1 | 206936049 | rs76325865 | T | 0.168 | 0.48 | *IL10* |
| 19 | 7810248 | rs11465381 | C | 0.230 | -0.31 | *CD209* |
