# Supplementary material for: Tuberculosis severity associates with variants and eQTLs related to vascular biology and infection-induced inflammation
Source: PLoS Genet. 2023 Mar 27;19(3):e1010387. doi: 10.1371/journal.pgen.1010387 (PMC10079228; doi:10.1371/journal.pgen.1010387)
Supplement: S11 Table — (DOCX) [file pgen.1010387.s012.docx]

**Table S11. Association with TBscore for SNPs within +/- 50Kb of *IL12B***

| **SNP** | **CHR** | **BP** | **BETA** | **L95** | **U95** | **P** | **Gene** | **Function** |
| --- | --- | --- | --- | --- | --- | --- | --- | --- |
| rs57803641 | 5 | 158780415 | -0.7168 | -1.364 | -0.0694 | 0.03164 | LOC285626 | Intron |
| rs13358509 | 5 | 158785247 | -0.7713 | -1.495 | -0.0473 | 0.03856 | LOC285626 | Intron |
| rs7722535 | 5 | 158792048 | -0.7713 | -1.495 | -0.0473 | 0.03856 | N/A | Intergenic |
