# Supplementary material for: Tuberculosis severity associates with variants and eQTLs related to vascular biology and infection-induced inflammation
Source: PLoS Genet. 2023 Mar 27;19(3):e1010387. doi: 10.1371/journal.pgen.1010387 (PMC10079228; doi:10.1371/journal.pgen.1010387)
Supplement: S14 Table — (DOCX) [file pgen.1010387.s015.docx]

**Table S14. Cohort Characteristics for Ugandan Subjects in Matrix eQTL Analysis**
