# Supplementary material for: Tuberculosis severity associates with variants and eQTLs related to vascular biology and infection-induced inflammation
Source: PLoS Genet. 2023 Mar 27;19(3):e1010387. doi: 10.1371/journal.pgen.1010387 (PMC10079228; doi:10.1371/journal.pgen.1010387)
Supplement: S17 Table — (DOCX) [file pgen.1010387.s018.docx]

**Table S17. rs2976562 Allele Frequencies in 1000G Project**

| **Population** | **Allele Frequencies** |
| --- | --- |
| **African** | **C: 88.7% T: 11.3%** |
| ACB | C: 83.3% T: 16.7% |
| ASW | C: 83.6% T: 16.4% |
| ESN | C: 88.9% T: 11.1% |
| GWD | C: 93.4% T: 6.6% |
| LWK | C: 85.4% T: 14.6% |
| MSL | C: 93.5% T: 6.5% |
| YRI | C: 90.3% T: 9.7% |
| **American** | **C: 42.4% T: 57.6%** |
| **East Asian** | **C: 25.1% T: 74.9%** |
| **European** | **C: 53.7% T: 45.3%** |
| **South Asian** | **C: 33.3% T: 66.7%** |
