# Supplementary material for: Tuberculosis severity associates with variants and eQTLs related to vascular biology and infection-induced inflammation
Source: PLoS Genet. 2023 Mar 27;19(3):e1010387. doi: 10.1371/journal.pgen.1010387 (PMC10079228; doi:10.1371/journal.pgen.1010387)

**Figure S3. Plot of PC1 vs. PC2 in Cohort 1.** PC1 vs. PC2 plots show the first two principal components of the genotype data, i.e. the ones that represent the greatest amount of variation in the data. Multiple distinct clusters would suggest the existence of sub-groups within the population in question. As we do not see any here, it does not indicate that there any genetically distinct sub-groups within our data. PC1 explains 2.8% of the variation and PC2 explains 2.6% of the variation. The fact that both of these percentages (especially for PC1) are so low also suggests that the principal components are not meaningful representations of sub-groups within the population.


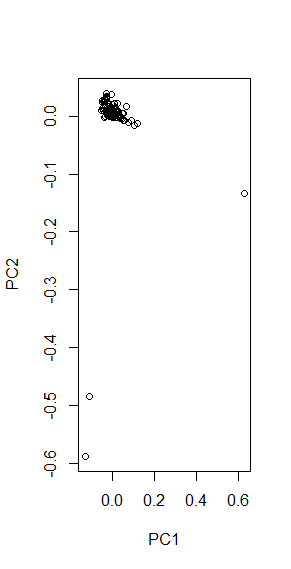

Supplement: S3 Fig — PC1 vs. PC2 plots show the first two principal components of the genotype data, i.e. the ones that represent the greatest amount of variation in the data. Multiple distinct clusters would suggest the existence of sub-groups within the population in question. As we do not see any here, it does not indicate that there any genetically distinct sub-groups within our data. PC1 explains 2.8% of the variation and PC2 explains 2.6% of the variation. The fact that both of these percentages (especially for PC1) are so low also suggests that the principal components are not meaningful representations of sub-groups within the population. (DOCX) [file pgen.1010387.s021.docx]
