# Supplementary material for: Tuberculosis severity associates with variants and eQTLs related to vascular biology and infection-induced inflammation
Source: PLoS Genet. 2023 Mar 27;19(3):e1010387. doi: 10.1371/journal.pgen.1010387 (PMC10079228; doi:10.1371/journal.pgen.1010387)
Supplement: S7 Fig — (DOCX) [file pgen.1010387.s025.docx]

**Figure S7. Categories for X-Ray severity**

0 = Normal

1 = Minimal disease -- infiltrates of slight to moderate density; disease may be present in a small portion of both lungs; the total volume of the

infiltrate(s) must be the volume of one lung present above the second chondrosternal junction and the spine of the fourth or the body of the

fifth thoracic vertebra; no cavitation may be present.

2 = Moderately advanced disease -- disease may be present in one or both lungs; the total extent must not be more than the following:

(a) scattered lesions of slight to moderate density may not involve more than total volume of one lung or the equivalent volume of both lungs.

(b) dense, confluent lesions may not involve more than 1/3 of the volume of one lung.

(c) the total diameter of cavity(ies) may not be greater than 4 cm.

3 = Far advanced -- lesions more extensive than moderately advanced.
