# Supplementary material for: Tuberculosis severity associates with variants and eQTLs related to vascular biology and infection-induced inflammation
Source: PLoS Genet. 2023 Mar 27;19(3):e1010387. doi: 10.1371/journal.pgen.1010387 (PMC10079228; doi:10.1371/journal.pgen.1010387)
Supplement: S8 Fig — The Manhattan plot shows the inverse log(10) of the p-values for the association between each SNP and CXR extent on the y-axis and the x-axis represent the physical location of each SNP on the chromosomes, which are in order from 1–22. (DOCX) [file pgen.1010387.s026.docx]

**Figure S8. Manhattan Plot for Meta-Analytic P-values for association between SNPs and CXR Extent.** The Manhattan plot shows the inverse log(10) of the p-values for the association between each SNP and CXR extent on the y-axis and the x-axis represent the physical location of each SNP on the chromosomes, which are in order from 1-22.

**
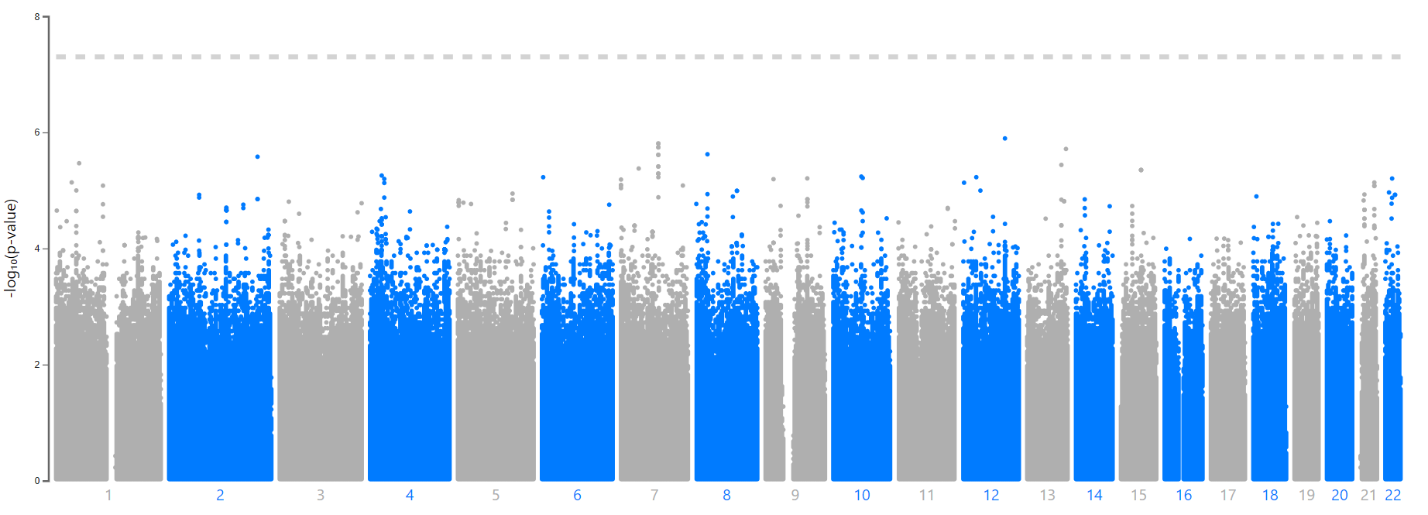
**
