# Supplementary material for: Tuberculosis severity associates with variants and eQTLs related to vascular biology and infection-induced inflammation
Source: PLoS Genet. 2023 Mar 27;19(3):e1010387. doi: 10.1371/journal.pgen.1010387 (PMC10079228; doi:10.1371/journal.pgen.1010387)
Supplement: S9 Fig — The quantile-quantile (Q-Q) plot shows the inverse log(10) of the observed p-values on the Y-axis relative to what is expected if there was no association on the x-axis. Deviations above the line indicate an association with the outcome. If the line deviates at the low quantiles, then this is considered evidence to suggest genome-wide inflation of the test statistics, which typically indicates unmeasured confounding. (DOCX) [file pgen.1010387.s027.docx]

**Figure S9. Q-Q Plot for Meta-Analytic P-Values for association between SNPs and CXR Extent.** The quantile-quantile (Q-Q) plot shows the inverse log(10) of the observed p-values on the Y-axis relative to what is expected if there was no association on the x-axis. Deviations above the line indicate an association with the outcome. If the line deviates at the low quantiles, then this is considered evidence to suggest genome-wide inflation of the test statistics, which typically indicates unmeasured confounding.

**
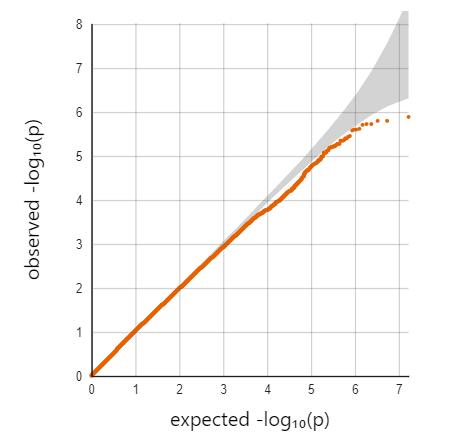
**
