# Supplementary material for: Tuberculosis severity associates with variants and eQTLs related to vascular biology and infection-induced inflammation
Source: PLoS Genet. 2023 Mar 27;19(3):e1010387. doi: 10.1371/journal.pgen.1010387 (PMC10079228; doi:10.1371/journal.pgen.1010387)

**Figure S10. Decile Regression between rs184553 and TBscore, adjusted for Sex and HIV.** The Y-axis shows the β value for the association between rs184553 and TBscore while the X-axis shows the quantiles of TBscore. Each dot on the line shows the β value at each decile of TBscore


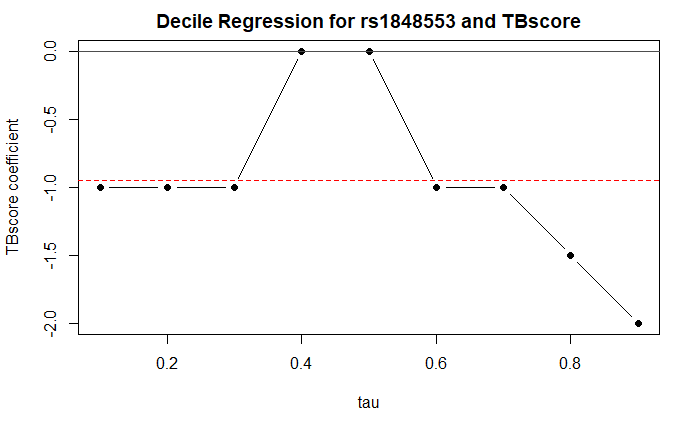

Supplement: S10 Fig — The Y-axis shows the β value for the association between rs184553 and TBscore while the X-axis shows the quantiles of TBscore. Each dot on the line shows the β value at each decile of TBscore. (DOCX) [file pgen.1010387.s028.docx]
