# Supplementary material for: Tuberculosis severity associates with variants and eQTLs related to vascular biology and infection-induced inflammation
Source: PLoS Genet. 2023 Mar 27;19(3):e1010387. doi: 10.1371/journal.pgen.1010387 (PMC10079228; doi:10.1371/journal.pgen.1010387)

**Figure S11. STRING Network for PPI’s from eQTL Response Genes.** This figure is a string diagram showing protein-protein interactions among the genes identified in my analysis. Lines represent an experimentally determined protein-protein interaction and multiple lines between the same two proteins indicates multiple interactions that have been identified, but multiple interactions between the same two proteins are still considered to be one edge.


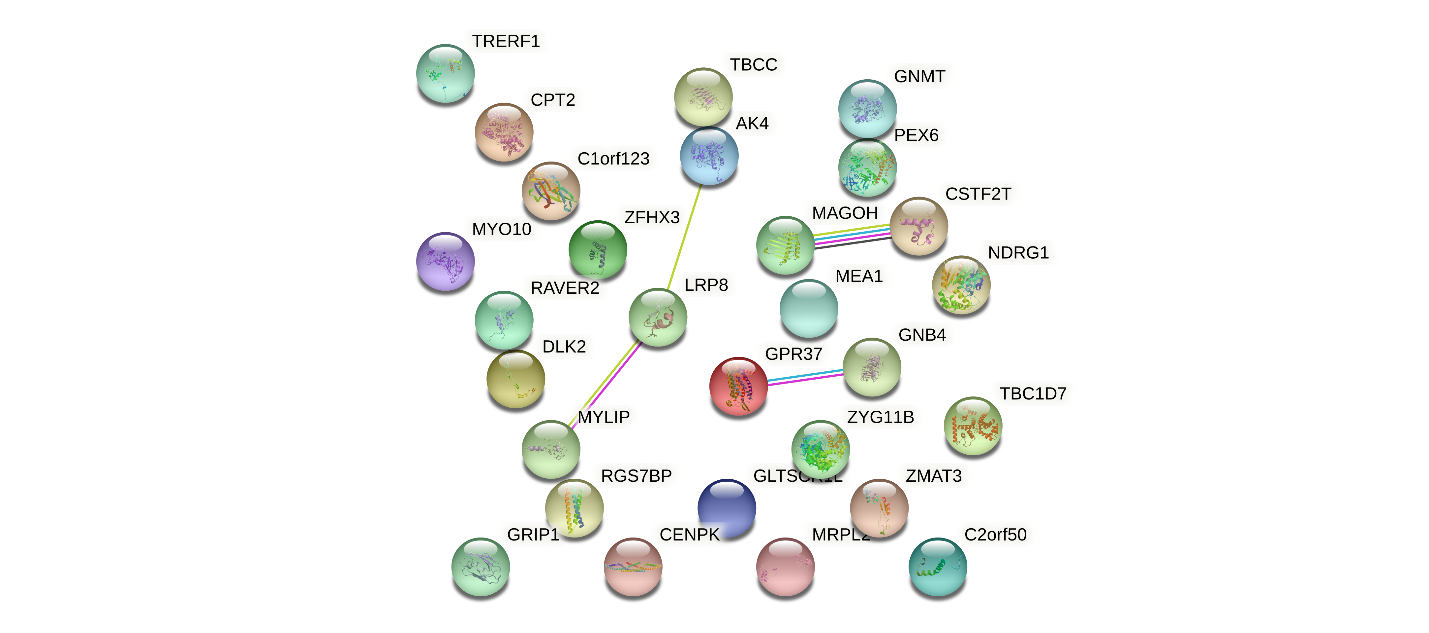

Supplement: S11 Fig — This figure is a string diagram showing protein-protein interactions among the genes identified in my analysis. Lines represent an experimentally determined protein-protein interaction and multiple lines between the same two proteins indicates multiple interactions that have been identified, but multiple interactions between the same two proteins are still considered to be one edge. (DOCX) [file pgen.1010387.s029.docx]
