# Supplementary figures and images for: Tuberculosis severity associates with variants and eQTLs related to vascular biology and infection-induced inflammation
Source: PLoS Genet. 2023 Mar 27;19(3):e1010387. doi: 10.1371/journal.pgen.1010387 (PMC10079228; doi:10.1371/journal.pgen.1010387)

**Figure S12. Expression of *SLA* in Immune Cells from DICE Database**


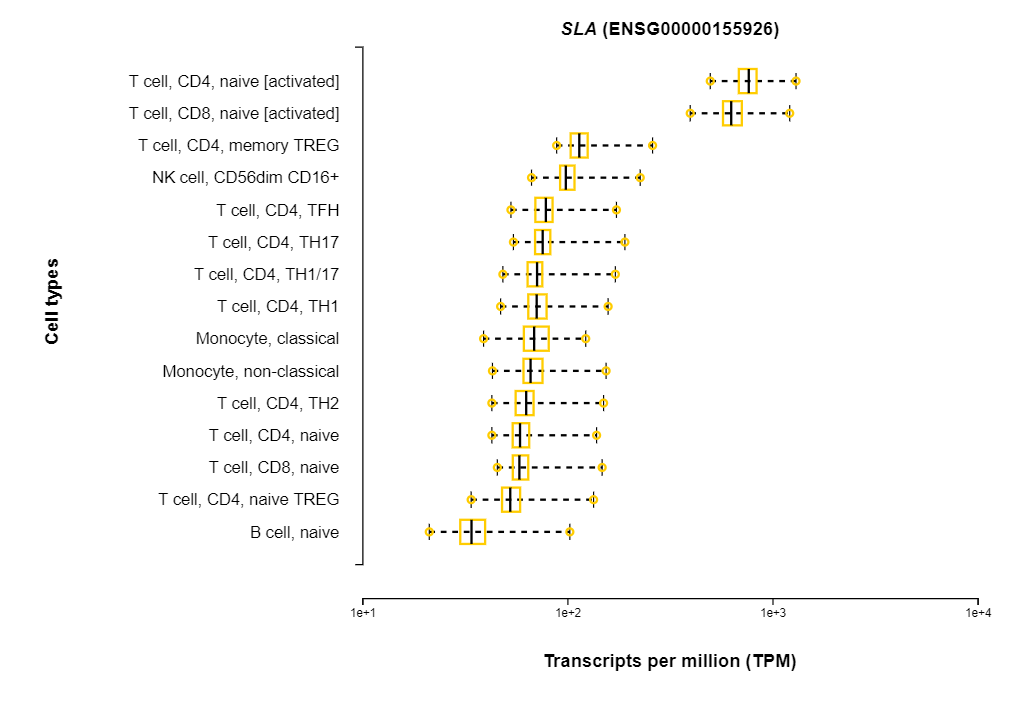

Supplement: S12 Fig — (DOCX) [file pgen.1010387.s030.docx]

**Figure S13. Imputation and QC of SNPs in Cohorts 1 and 2**

**
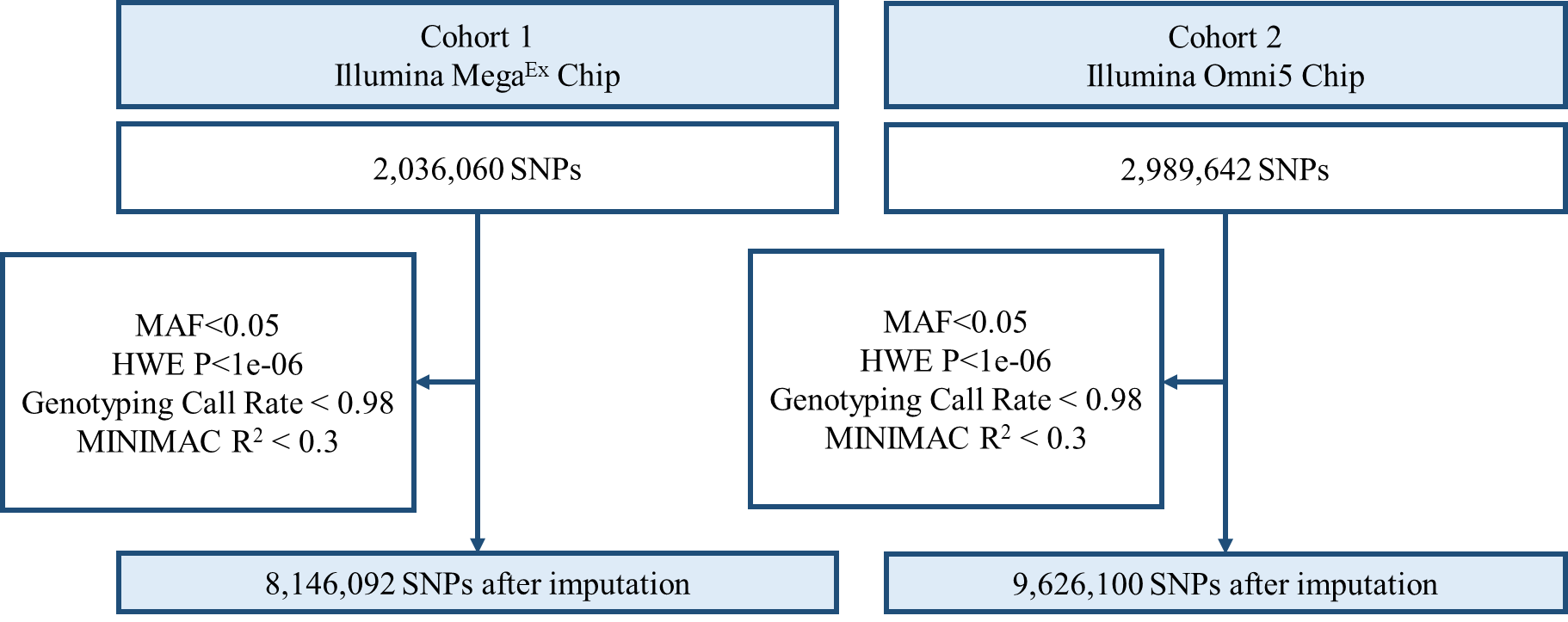
**

Supplement: S13 Fig — (DOCX) [file pgen.1010387.s031.docx]
